# Shelby County Medical Society

**Published:** 1859-02

**Authors:** Wm. Headen, L. D. Martin

**Affiliations:** President; Shelbyville; Secretary; Shelbyville


### SHELBY COUNTY MEDICAL SOCIETY.

SHELBYVILLE, December 21st, 1858.

According to previous notices, a meeting was held at the Masonic Hall, in Shelbyville, on the 21st, at 1 o'clock P.M., for the purpose of forming a Medical Society.

On motion, Dr. Wm. Headen was called to the chair, and Dr. L. D. Martin was chosen Secretary.

On motion of E. S. Penwell, Drs. Martin, E. Penwell, and J. W. York, were chosen as a Committee to draft a Constitution for the Society.

The Committee presented the draft of a Constitution, which was adopted, as follows, to wit:

*Article I.*—This Society shall be known as the SHELBY COUNTY MEDICAL SOCIETY.

*Article II.*—The officers of this Society shall be a President, Vice-President, Secretary, Treasurer, and Board of Censors, consisting of three persons.

*Article III.*—The duty of the President shall be to preside at all regular meetings of the Society; and to deliver an address before the Society at its first semi-annual meeting in each year, which address shall be *public.*

*Article IV.*—The duty of the Vice-President shall be to preside at the meetings of the Society, in the absence of the President; and deliver a public address before the Society at its second meeting in each year.

*Article V.*—The duty of the Secretary shall be to keep a record of the proceeding of the Society, and prepare the same for publication; also to preserve a copy of all essays which shall be read before the Society.

*Article VI.*—The duty of the Treasurer shall be to receive all moneys due the Society, and disburse them as per direction of the same.

*Article VII.*—It shall be the duty of the Board of Censors to examine all applicants for membership, as to qualifications, both professionally and as gentlemen, and duly report the same to the Society.

*Article VIII.*—The physicians present at the adoption of this Constitution, shall be constituted members of the Society.

*Article IX.*—This Society shall hold a semi-annual meeting; one on the 13th day of May, and one on the 18th day of November, in each year.

*Article X.*—Each member shall pay into the treasury, each year, at the first semi-annual meeting, $5, to be used at the discretion of the Society.

*Article XI.*—It shall be the duty of each member to prepare a paper, upon some subject of interest to the profession, to be read before the Society at each regular meeting.

*Article XII.*—This Society shall be governed by the code of ethics adopted by the American Medical Association.

*Article XIII.*—This Constitution may be altered or amended by a vote of the majority of members present at any regular meeting.

The following persons became members, under the provisions of Article VIII. of the Constitution:

| | |
|---|---|
| Dr. E. S. Penwell, | Dr. Enos Penwell, |
| " Wm. Headen, | " Eli York, |
| " J. W. York, | " Wm. M. Osborn. |
| " L. D. Martin, | |

On motion, the Society then proceeded to the election of officers for the ensuing year. The election resulted in the choice of the following officers:

| | |
|---|---|
| *President*, . . . . . | Dr. Wm. Headen. |
| *Vice-President*, . . | " J. W. York. |
| *Secretary*, . . . . | " L. D. Martin. |
| *Treasurer*, . . . . . | " E. S. Penwell. |
| *Board of Censors*, . . | " Eli York,<br>" Penwell,<br>" W. Osborn. |

On motion, each member was permitted to make choice of a subject upon which to write an essay, to be read at the next regular meeting. The subjects chosen were as follows:

| | |
|---|---|
| *Dysentery*, . . . . . | Dr. J. W. York. |
| *Milk-Sickness*, . . . . | " L. D. Martin. |
| *Typhoid Fever*, . . . . | " E. S. Penwell. |
| *Typhoid Pneumonia*, . . | " Wm. M. Osborn. |
| *Scarlet Fever*, . . . . | " Eli York. |

A motion was then made that the proceedings of the meeting be published in the *Chicago Medical Journal* and *Okaw Patriot.*

On motion, the Society then adjourned to meet in Shelbyville, on the 13th day of May, 1858, at 10 o'clock A. M., the place to be hereafter designated.

WM. HEADEN, M.D., *President.*

L. D. Martin, M.D., *Secretary.*

